# Implementation of clinical practice changes in the PICU: a qualitative study using and refining the iPARIHS framework

**DOI:** 10.1186/s13012-021-01080-9

**Published:** 2021-01-28

**Authors:** Katherine M. Steffen, Laura M. Holdsworth, Mackenzie A. Ford, Grace M. Lee, Steven M. Asch, Enola K. Proctor

**Affiliations:** 1 Department of Pediatrics, Division of Pediatric Critical Care Medicine, Stanford University, 770 Welch Road, Suite 435, Palo Alto, CA 94304 USA; 2 Stanford Division of Primary Care and Population Health, Stanford, CA USA; 3 Department of Pediatrics, Division of Pediatric Cardiology, Stanford University, Palo Alto, CA USA; 4 Department of Pediatrics, Division of Pediatric Infectious Diseases, Stanford University, Palo Alto, CA USA; 5 VA Center for Innovation to Implementation, Stanford Division of Primary Care and Population Health, Palo Alto, CA USA; 6 George Warren Brown School of Social Work, Washington University in Saint Louis, Saint Louis, MO USA

**Keywords:** Clinical practice change, Implementation, Intensive care unit, Pediatric

## Abstract

**Background:**

Like in many settings, implementation of evidence-based practices often fall short in pediatric intensive care units (PICU). Very few prior studies have applied implementation science frameworks to understand how best to improve practices in this unique environment. We used the relatively new integrated Promoting Action on Research Implementation in Health Services (iPARIHS) framework to assess practice improvement in the PICU and to explore the utility of the framework itself for that purpose.

**Methods:**

We used the iPARIHS framework to guide development of a semi-structured interview tool to examine barriers, facilitators, and the process of change in the PICU. A framework approach to qualitative analysis, developed around iPARIHS constructs and subconstructs, helped identify patterns and themes in provider interviews. We assessed the utility of iPARIHS to inform PICU practice change.

**Results:**

Fifty multi-professional providers working in 8 U.S. PICUs completed interviews. iPARIHS constructs shaped the development of a process model for change that consisted of phases that include planning, a decision to adopt change, implementation and facilitation, and sustainability; the PICU environment shaped each phase. Large, complex multi-professional teams, and high-stakes work at near-capacity impaired receptivity to change. While the unit leaders made decisions to pursue change, providers’ willingness to accept change was based on the evidence for the change, and provider’s experiences, beliefs, and capacity to integrate change into a demanding workflow. Limited analytic structures and resources frustrated attempts to monitor changes’ impacts. Variable provider engagement, time allocated to work on changes, and limited collaboration impacted facilitation. iPARIHS constructs were useful in exploring implementation; however, we identified inter-relation of subconstructs, unique concepts not captured by the framework, and a need for subconstructs to further describe facilitation.

**Conclusions:**

The PICU environment significantly shaped the implementation. The described process model for implementation may be useful to guide efforts to integrate changes and select implementation strategies. iPARIHS was adequate to identify barriers and facilitators of change; however, further elaboration of subconstructs for facilitation would be helpful to operationalize the framework.

**Trial registration:**

Not applicable, as no health care intervention was performed.

**Supplementary Information:**

The online version contains supplementary material available at 10.1186/s13012-021-01080-9.

Contributions to the literature

- This analysis identifies common barriers and facilitators of practice change in the PICU, which may inform implementation of future evidence-based interventions in this care setting.
- Limited research has applied the iPARIHS framework, although the parent PARIHS is widely cited. This study adds to the literature by using this new framework to address implementation planning and strategy selection in the PICU.
- We noted limitations of the iPARIHS framework, particularly around the facilitation construct, but provide descriptions of useful activities that may improve understanding and specification of facilitation.

## Background

Advances in care for critically ill children have improved outcomes. However, too often modern medical care trades reduced mortality for increased morbidity in pediatric intensive care units (PICUs) [1]. One contributing reason is that existing evidence-based practices are often not used as frequently as recommended. As in many health care settings, implementation of evidence in the intensive care unit (ICU) presents challenges, some of which may be due to patient complexity, stress, and an increased risk for error [2]. Moreover, specific challenges to implementation related to the pediatric population and environmental context of the PICU may also exist.

Adult ICU studies provide some insight into ICU-specific implementation issues, but often focus on implementation of a specific intervention [3–7] such as post-arrest care [8], ICU care bundles [9], or handoffs [10], or utilize a specific implementation approach [2, 5, 11]. Prior studies report that identifying and addressing local, contextual implementation barriers may help to optimize care delivery in the ICU [12–14], which include lack of awareness of the innovation/guideline [7, 15], lack of resources [11], competing priorities [11], apprehension around change [7], poor formatting of the innovation for the clinical context [7], and inability to provide accurate feedback on performance [11]. Sinuff et al. noted a fundamental need for an ICU culture to support a new intervention [16]. Other studies identified engaging staff, education, providing adequate time and resources, reminders, audit and feedback, and data reporting on performance as being useful implementation strategies [5, 6, 8, 9, 15, 16], with providers in different roles reporting different implementation strategies most useful in effecting change [15].

While adult ICU studies may begin to inform implementation of clinical practice changes in the PICU, to our knowledge, no study has attempted to characterize unique PICU barriers and facilitators. We sought a framework that would account for the complexity of this environment and identified the integrated Promoting Action on Research Implementation in Health Services (i-PARIHS) framework [17]. This framework and the parent Promoting Action on Research Implementation in Health Services (PARIHS) acknowledge complexity of implementation in actual practice. PARIHS is a widely used conceptual framework designed to explain or predict success of implementation [18, 19]. i-PARIHS differs from PARIHS in that it incorporates a broader view of evidence and context, acknowledges the function recipients play in implementation success, and places further emphasis on the role of facilitation [17]. In iPARIHS, the innovation, recipients, and context exist as constructs that are modified by facilitation in the process of implementation [17].

This qualitative study aimed to address a gap in the literature around PICU-specific implementation barriers and facilitators using the i-PARIHS framework and puts forth a comprehensive process model to understand change in the PICU. Rather than focusing on a single intervention, we broadly explore implementation in this complex system. This approach has the potential to inform adoption, delivery, and sustainability of different types of evidence-based interventions and translate to implementation benefits across the PICU population [20]. Additionally, we provide insight into using the relatively new i-PARIHS framework and comment on utility in this clinical setting.

## Methods

### Study design

Semi-structured qualitative interviews were carried out to prepare for a study that will examine prospective implementation of new blood transfusion recommendations for critically ill children. The interviews aimed to understand prior experiences with implementing clinical practice changes within the PICU. Ethics approval for the study was obtained from the Stanford University Institutional Review Board (IRB-47140). The completed Standards for Reporting Qualitative Research (SRQR) checklist https://www.equator-network.org/reporting-guidelines/srqr/ is included as additional file 1.

### Participants and setting

We conducted interviews with health care providers working in various roles from eight PICUs across the USA. We selected units to represent variation in PICU types (pediatric ICU (excluding cardiac patients, 4 units), pediatric cardiovascular ICU (CVICU) (2 units), and combined pediatric/cardiovascular PICU/CVICU (2 units)) and overall PICU size (11-32 beds) in the USA. The three types of ICUs are collectively referred to as PICUs, unless specified. A unit representative helped recruit participants. The study team and unit representative sent an initial e-mail to solicit volunteers for participation. We provided additional information about the study to respondents and contacted them if they agreed to participate following review of this information. We selected participants using a stratified, purposeful sampling strategy, as described by Palinkas [21] to achieve a sample of participants within each PICU role. One of two authors (KS and MF) conducted interviews either in person or via telephone. We obtained the participant’s verbal consent prior to each interview. Interviews were audio recorded and transcribed verbatim. We continued interviewing participants in each role until no new information was forthcoming from new participants. The qualitative team members (KS, LH, and GL) held meetings to review data and confirm when variation in responses was no longer noted.

We developed an interview topic guide from the iPARIHS framework [17]; however, added topics that focused on barriers and facilitators of clinical practice changes and blood transfusion in the intensive care unit to ensure we addressed aspects of change that are not explicit in the iPARIHS framework (Additional file 2). Participants were asked first to describe a clinical practice change that had been implemented in their ICU as a starting point to discuss the process and impact of these initiatives. Additional questioning focused on exploring PICU culture, receptivity to clinical practice change, initiating and sustaining practice changes, and experiences and perspectives on blood transfusion in the PICU. Qualitative data related to blood transfusion is not reported in this manuscript.

### Qualitative analysis

We used a Framework Approach [22] for qualitative analysis with the NVivo (Version 12) software package. Framework is an approach to analysis in which case-level data (rows) are summarized along thematic categories (columns) in a matrix and involves five steps of familiarization, identifying a thematic framework, indexing, charting, and mapping and interpretation [23]. The coding framework was structured around the iPARIHS constructs (innovation (the evidence-based intervention), recipients, context, and facilitation) with associated subconstructs [17]. We applied iPARIHS in this specific setting to gain insight into the combination of factors required for successful implementation in this clinical care environment [20] and move toward precision implementation to ultimately better specify and tailor implementation strategies [24]. Subconstruct definitions were not available to standardize analysis; therefore, we developed a codebook (Additional file 3) to define subconstruct items using definitions drawn from the revised PARIHS framework [25], the Consolidated Framework for Implementation Research [26], and the Theoretical Domains Framework [27]. The *facilitation* construct was coded broadly to include any data wherein deliberate support or problem solving was applied to promote implementation. Following coding, review, and organization of data coded within the facilitation construct, it became evident that this data broadly aligned with the implementation strategies as described in the Expert Recommendations for Implementing Change (ERIC) project by Powell et al. [28]; thus, we organized this data within the ERIC categories. We also added inductive codes while searching for emergent themes, particularly around implementation barriers and facilitators to address potential constraints of using the iPARIHS framework to structure our analysis. At the outset of the analysis, two researchers (KS, LH) selected and coded fived interviews independently using the a priori codes from iPARIHS, while creating new codes for emergent themes. The researchers met to agree on definitions and interpretations of existing codes, compare coding, and discuss emerging themes and integrate into the coding framework. One researcher (KS) coded the remainder of the interviews using the established coding strategy. We initially categorized coded data within the iPARIHS subconstructs, then, following the Framework approach, summarized data to retain context for ease of comparisons across units and roles. Following summarization, we compared and contrasted data between roles and clinical units, with careful consideration of identifying patterns as well as unique, but informative statements that provided an alternative perspective to capture variation. Strategies to ensure credibility (internal validity) of findings followed guidance by Miles et al. [29] and included linking data to categories in iPARIHS, checking for negative evidence, and checking that findings are replicable across the dataset (i.e., across more than one PICU).

## Results

Fifty health care providers were interviewed: PICU attendings (*n* = 15), fellow trainees (6), resident trainees (4), nurse practitioners (NPs) or physician assistants (9), nurses (10), and subspecialty physicians/surgeons whose patients were cared for regularly in a PICU (6), such as hematologists/oncologists, cardiologists, and general and cardiothoracic surgeons. Providers in each role were interviewed at each site, with the exception of fellow or resident trainees, as they were not present in some units. Unless specified, the term “providers” refers to these individuals collectively. Interviews lasted a median of 53.5 min (range = 37–79 min) and were carried out between December 2018 and June 2019. Through exploration of the iPARIHS framework’s *innovation*, *recipient*, *context*, and *facilitation* constructs, we identified six themes and nine sub-themes that were important for change in the PICU.

### The PICU as a high-risk environment for clinical practice change

Within the iPARIHS *recipients* and *context* subconstructs, providers identified unique sub-themes of the PICU environment that increased complexity around clinical practice change and had important implications for implementation. These include added complexity with team-based care, high-stakes care that limited receptivity to change, variable readiness for change, limited bandwidth, and the emotional toll of the PICU environment; each sub-theme is further described in Table 1. Of note, receptivity to change related to providers being amenable to or interested in change conceptually, whereas readiness to change occurred when providers had the appropriate resources (time, skills, support) to operationalize the change. When providers were receptive to or ready for a change to occur, change was facilitated. Conversely, when providers were either not receptive to a change or not ready to integrate the change into practice, this served as a barrier to change.

**Table 1:** Description of sub-themes with examples

iPARIHS construct (s)	iPARIHS sub-construct (s)	Theme	Sub-theme	Description	Example
Recipients Context	Collaboration and teamwork Local level: Mechanisms for embedding change	PICU as a high-risk environment for clinical practice change (section 3.1)	Complexity with team-based care	Care is provided by multi-professional teams, each with different knowledge and expertise. Change required seeking out input, ensuring buy-in, or providing information to all teams, adding complexity	“whenever you want to actually effect any kind of significant change, there’s a lot of people who have to get on board. And of course, as soon as you add more cooks in the kitchen things seem to slow down” (Site 1 CVICU, Attending 1).
Recipients Context	Motivation Local level: Culture		High-stakes limit receptivity to change	PICU care and decision-making is high-risk, with potential for significant morbidity or mortality. Providers described a desire to avoid negative outcomes at all costs.	“Our culture is that people like to use the same protocols because they’re proven, and not try to change…especially when it comes to babies’ health” (Site 2 CVICU, surgical subspecialist 34).
Recipients Context	Values and beliefs; Motivation Local level: Culture		Variable readiness for change	Continuous changes in patient status and need for alterations in care plans make some providers ready to change, and some resistant to it.	“People who work in ICU tend to be kind of constantly ready for a change in patient condition, they are also kind of hard wired or prepared for changes in other ways” (Site 3 PICU, NP 23) “The unit is a …very intense and chaotic place. I think we crave stability and some of the stability is centered around easy decision making.” (Site 2 Combined PICU/CVICU, Attending 44)
Recipients Context	Time, resources, support Local level: Culture		Limited bandwidth	Units often functioned at limits of capacity; providers felt overworked, reported units were understaffed. Staff and leadership turnover and temporary staff (i.e. traveling nurses, resident trainees) posed challenges to consistent use of new practice changes.	“Inertia from being overworked is a barrier [to change]. People have really high clinical loads, so they don’t want to make a change. If it's not already part of your habit…gets pushed down the priorities” (Site 3 PICU, Fellow 22)
Context	Local level: Culture		Emotional toll of the PICU environment	Emotional, physical and mental exhaustion noted due to unpredictable and difficult cases. Emotions influenced efforts to change practice.	“Most kids do not have any of the procedures or outcomes that our patients do. I think that definitely flavors how people react to things... I think the stakes are higher in many ways for many of the things that we do, or at least it feels that way.” (Site 1 Combined PICU/CVICU, Attending 9).
Innovation	Underlying knowledge sources	Individual Determinants (section 3.4)	Evidence for change	Strong scientific evidence for change was a powerful stimulus to convince providers to change, particularly for physicians and NPs.	“For providers – is it clinically relevant, does it provide a benefit? How much? We need to see research. We need to see data that shows that this is going to be worth the effort.” (Site 3 PICU, NP 24)
Innovation Recipients	Relative advantage Values and beliefs; Motivation		Rationale for change	Rationale: Understanding the rationale for a change, goals, and potential benefits of a change were vitally important for all providers.	“Sometimes people don’t fully understand the reason for trying to make a change… Either that it’s not relayed or explained in the right manner…if there was a better understanding they'd be more likely to be on board with it.” (Site 1 Combined PICU/CVICU, Attending 11)
Recipients	Values and beliefs; Motivation		Provider level factors: provider experience	Apprehension around change: Providers noted being apprehensive about change, preferring the “old way to do things”	“The hardest thing [about change] is people are just so used to doing it a certain way. It makes people nervous to change the way that they’ve always done things. It may take you out of your comfort zone, and people don’t like that.” (Site 4 PICU, NP 35)
Recipients	Motivation; skills and knowledge			Duration of Experience: More senior providers were often identified as being less receptive to change than less senior providers, however these providers also influenced practice and could facilitate acceptance of change.	“The people who have been here for the longest, are the least likely to embrace change…The early adopters are the younger people. Especially the people who come from other places and who have seen it done differently.” (Site 1 CVICU, Attending, 2)
Recipients	Values and beliefs; Motivation		Provider level factors: beliefs	Perceived need: Change was perceived as “needed” if 1) the existing process was frustrating, 2) the change prevented a bad outcome, or 3) it aligned with the provider’s priorities, hospital values, or practices at other centers (benchmarking).	“I think [the change was easy because] everyone was a little bit frustrated with the lack of process, or protocol previously” (Site 1 PICU, Attending 6)
Recipients	Values and beliefs; Motivation; Skills and knowledge			Potential for negative outcome: Providers worried changes might have a negative impact on outcomes and new practices may be less safe in inexperienced hands. Significant shifts in practice were more difficult to accept.	“It’s not just that we’re stuck in our ways. We want to do things that we know we’re good at and that are effective. Even if something [new] might be slightly more safe, is it more safe in my hands? I'm not sure.” (Site 1 PICU Attending 5)
Recipients	Values and beliefs			Compromised autonomy: Some providers felt creation of standard practices might compromise their autonomy.	“Some people feel like they’re getting their freedom taken away…they aren’t going to have that creative freedom to choose how they want to manage a patient... when they feel like their overall authority is being taken away, it’s hard for some people” (Site 4 PICU, NP 35)
Recipients	Values and beliefs; Motivation		Provider level factors: Perceived benefit/effort	Cost vs. benefit of change: Many changes require additional time or adding a task; the benefit of the change must exceed the “cost” of changing, otherwise it is less likely to be accepted or performed consistently.	“There’s probably some balance of how important is [the change]? How easy is it? If something’s really important, even if it’s really difficult to do, people will do it. If something’s only moderately important then you probably need a low amount of resistance to do it.” (Site 2 CVICU, Fellow 29)
Recipients Context	Time, resources, support Local level: Culture		Competing interests and time	Changes that were unrealistic due to other demands/tasks were unlikely to be supported or carried out.	“It’s hard to keep adding all of these changes. We already do so much during a 12-h period. To add something else is just, “Why are we doing “one more thing”.” (Site 1 Combined PICU/CVICU, Nurse 13)

### Three sources of change

Providers reported that the stimulus for change in the PICU, located within iPARIHS *innovation* and *context* constructs, typically originated from three sources: (1) from a motivated individual or group with a specific interest working within the unit, (2) from a source outside the unit, or (3) as a result of a negative or sub-optimal clinical outcome (iPARIHS Construct: *Context*; Sub-constructs: *Local level Culture*, *Local level Past experience with innovation and change*, *Organizational level Priorities*, *Organizational level Culture*). Based on prior experience or personal interest, individuals or groups proposed change initiatives to improve care. Outside sources included the hospital, hospital system, or, infrequently, a regulatory agency; associated changes were usually intended for the entire hospital or organization. Often, these changes did not account for PICU workflow or unit dynamics and were more difficult for providers to both accept and integrate into practice (iPARIHS Construct: *Innovation*; Sub-constructs: *Degree of fit with existing practice and values*). Providers disliked these changes “imposed” on the PICU and preferred when they were modified to fit PICU practice. Providers of all professions noted that changes were made in response to adverse outcomes with significant or unexpected morbidity or mortality (iPARIHS Construct: *Context*; Sub-constructs: *Local level Culture*, *Local level Past experience with innovation and change*). In these cases, clinical practices that were felt to be causal were often modified or abandoned despite prior demonstration of safety or success as providers were highly motivated to avoid future negative events: “There’s a lot of anecdote as well. Like there was that one bad case one time, so we don’t ever want to try that again” (Site 1 CVICU, Attending 2). Changes made in response to adverse outcomes were often made quickly and sometimes without obtaining provider input.

### Unit leadership and unit-based processes: decision to make a change guided by leadership

Prior to initiating change, unit leaders made the decision to devote resources and pursue a change. Identified through the iPARIHS *recipients* and *context* constructs, there was variation in who made the decision to pursue a change in practice. PICU physician and nursing leaders were often noted to work together to decide on making changes; however, in some cases, nursing or physicians made decisions independently without input from other professions (iPARIHS Constructs: *Recipients*, *Context*; Sub-constructs: *Power and authority*, *Local level Culture*). One NP noted: “A lot of policies and changes to nursing care are driven by nursing leadership. The medical team leadership is not necessarily involved in those changes…[it’s problematic because] we don’t learn about new standards of care until the nurse actually prints out a new copy of it” (Site 1 Combined PICU/CVICU, NP 12). Half of units had unit-based committees (multi-professional or nursing only) that informed leadership’s decision to make a change. Other professional groups (respiratory therapists, etc.) and subspecialists were routinely omitted in many units from the decision to make a change, though there was a broad understanding that obtaining feedback, making modifications, and obtaining buy-in from all professions as change moved forward was essential (iPARIHS Construct: *Context*; Sub-constructs: *Local level Culture*, *Local level Past experience with innovation and change*).

### Individual determinants: multiple determinants impacted a provider’s decision to accept and adopt change

While unit leaders made the decision to institute a change in the unit, in many cases, individual providers made an independent decision to support the change as part of their practice. While some changes were noted to be mandatory, providers who did not support a change could avoid using it in many cases, as there was not direct accountability. The individual provider’s impression of a proposed change impacted their willingness to comply with or resist the change. This impression was determined by four sub-themes within different iPARIHS constructs: (1) the evidence behind the change (iPARIHS Construct: *innovation*; Sub-constructs: *Underlying knowledge sources*), (2) rationale for change (iPARIHS Constructs: *innovation*, *recipients*; Sub-constructs; *Relative advantage*, *Values and beliefs*; *Motivation*), (3) provider-level factors (iPARIHS Construct: *recipients*; Sub-constructs: *Values and beliefs*; *Motivation*; *Skills and knowledge*), and (4) competing interests and time (iPARIHS Constructs: *recipients*, *context*; Sub-constructs: *Time*, *resources*, *support*; *Local level Culture*) (Table 1). Provider level-factors were intrinsic to the individual provider that influenced perception of and receptivity to change. We identified three factors within this sub-theme: provider experiences, provider beliefs, and balancing benefit with effort. Provider experiences refer to how receptivity to change and implementation were dependent on a provider’s level of experience or prior experiences working in the PICU. Provider beliefs were ideas providers held about their own practice or change in the PICU that impacted their receptivity to change. Finally, all providers perceived that change required an additional investment of time and effort. Each provider explicitly or implicitly recognized that they balanced the “costs” of a change (time, effort) with the perceived benefits of the intervention. Table 1 describes each of these sub-themes and factors in detail and provides an example of how providers discussed them.

### Operationalization of facilitation through implementation strategies

We gained perspective on the iPARIHS *facilitation* construct by asking providers about barriers and facilitators of change as well as prior experiences with change. Broad coding for this construct yielded a wide range of data elements describing actions and strategies used to promote implementation. These data were often linked with one or more of the *innovation*, *recipients*, and *context* constructs, which made it difficult to characterize elements within a single iPARIHS construct given the unique PICU context. The actions and strategies that constituted facilitation mostly fit within the ERIC categories of planning, restructuring, education, quality management [30], as detailed in Table 2. These facilitation strategies have key features that are unique to the PICU environment which we will describe.

**Table 2:** Implementation strategies important for facilitation in the PICU

Strategy	Description	Example
**Plan strategies**		
Tailor strategies to overcome barriers and honor preferences^a^	Tailor strategies to overcome barriers and honor preferences	“I think engagement^c^ of the bedside providers [helps change occur]...really trying to understand how a change will affect the work and then modify the proposed change based on the information that you get from people at bedside.” (Site 3 PICU, Attending 21)
Conduct local consensus discussion^a^	Achieving buy-in from leadership and bedside providers, creating ownership around change for those who use change on a daily basis	“Giving more ownership to the bedside nurses about the changes that are impacting the practice helps change to be a little bit easier.” (Site 1 Combined PICU/CVICU, Nurse 13)
Identify and prepare champions^a^	Identifying champions from each discipline impacted by change	“Having people who are champions at multiple different levels of stakeholders…whoever it may be that’s going to be involved to really lead that effort helps change occur” (Site 4 PICU, Attending 36)
**Restructure strategies**		
Change physical structure, equipment, records systems^a^	Embedding change into work systems (including EMR)	I think electronic medical record is probably the one thing that can help a lot with [implementation] since there is so much of our work [that’s done there] (Site 4 PICU, Fellow 38)
**Educate strategies**		
Conduct educational meetings^a^	Multi-professional education around change	“I think that there would have to be education [for a proposed change]. And not just for physicians, but also for nurse practitioners and for bedside nurses to understand why and where this is coming from” (Site 1 CVICU, Attending 2)
Make training dynamic^a^	Creating ways to reliably inform large PICU teams about change (using multiple modes of communication)	I think it is helpful doing [education] in a multitude of ways that are kind of repeated, to capture as many people as possible. (Site 4 PICU, Attending 36)
Time-sensitive training	Just in time education around the time of change roll-out	“For nurses, [change is facilitated by] “boots on the ground” things. The huddles, just -in-time education. (, more responsive to that sort of environment. (Site 2 PICU, Attending 20)
Develop effective educational materials^a^	Creating easy access to information about change, tools needed	“[a new protocol/process] would need to be in a place of easy access… in academic institutions where you’re having new rotators come through every several weeks, [you need to] make sure that there is a way to introduce it to folks” (Site 2 Combined PICU/CVICU, Resident 50)
Advance notice about change	Giving providers time to prepare for change	“[Change is hard if] it really greatly impacts our workflow and doesn't come with any preparation…If I know it’s coming, and I can personally think about how is this is going to affect my practice, and how I’m going to mitigate that issue (Site 3 PICU, Nurse 25)
Practice using change	Repeated use of change to create “muscle memory” around change (infrequently used changes are much harder to incorporate)^b^	“It’s repetition [that helps change “stick”]. Whether it’s repetition of practice or just repetition of education …sometimes everybody agrees that there’s going to be a policy change but it may be six months before you encounter a situation where that change would be implemented and then nobody remembers” (Site 2 PICU, medical subspecialist 18)
**Quality management strategies**		
Develop and organize quality monitoring systems^a^/Audit and provide feedback^a^	Tracking data/evaluation of the impact of change (monitoring compliance, outcomes, safety data)^b^	I think reporting back…so that people know, “Hey this thing we started this nine months ago?...Here’s what happened. That’s obviously an incentive for people to feel like it made a difference” (Site 2 CVICU, Attending 28)
Remind clinicians^a^	Frequent reminders about existence, importance of/rationale behind new change^b^	“[change is facilitated by] Informal reminders…the way sometimes people will remind you, “All right, let’s make sure we are doing our check lists at the end of every patient,” or the way people say, “let’s remember to wash our hands in and out of every room.” (Site 2 CVICU, Fellow 29)
Conduct cyclical small tests of change^a^	Iterative changes made to enhance use of change process	“[to make change “stick”] a check-in and a feedback session would probably be good, and if small changes need to be made to whatever protocol that’s established, they can be made and re-evaluated.” (Site 2 PICU, Attending 17)
**Other strategies**		
Ensuring adequate resources	Obtaining adequate resources to plan, implement, and sustain change	“Resources [can be a barrier to change] as well. Most changes require some sort of time, effort, if not other financial resources to implement.” (Site 4 PICU, Attending 36)
Communicating early success	Celebrating “successes” of change	“One of the ways that we found [to help make change] most successful was celebrating our successes…we put it in the newsletter, and celebrated it.” (Site 3 PICU, Nurse 25)
Accountability	Creating accountability for change (may be from frontline staff)	“There needed to be more accountability for…how do we make sure these things get done? So, they developed this little list…you had to sign off, you had to turn it in before you left. That went on for 6 months, and then people had kind of built a habit into their practice.” (Site 2 Combined PICU/CVICU, Nurse 48)

^a^Denotes described ERIC strategy

^b^Noted to be important for sustainability of change

^c^Engagement in this case denotes the process in which a those planning a change solicit feedback from providers who will use a change to provide insight into how to optimize implementation

Sub-optimal levels of engagement around new evidence and need for practice change (iPARIHS *recipients*, *context*) among some providers were an important barrier to changing existing clinical practice. This often stemmed from professional expectations and requirements. Nurses were not expected to stay abreast of new evidence which impacted receptivity to change, as described by one nurse: “Not all the nurses have made it part of their professional practice to stay updated on evidence… Practice changes that come from new evidence can be a little more challenging for that group” (Site 3 PICU, Nurse 25). Support given to nurses and other non-physician providers to spend time working on practice changes was also limited: “One of the challenges is that they [hospital administration] call our work [on change] ‘non-productive time’…they don’t allot a lot of resources to it. Some people are willing to go above and beyond without getting paid, but it’s not very well resourced” (Site 3 PICU, Nurse 25). As such, lack of expectations or incentives to work on change in these roles hindered progress.

Providers felt identifying a champion within each professional role was necessary to ensure a resource existed to support each group (iPARIHS *recipients*, *context*). Champions were stated to be knowledgeable about and promote use of the change, remind and reinforce why change was being made, and in some cases, assist with implementation. As in many implementation efforts, achieving buy-in from both leadership and front-line staff was essential; in addition, for nurses, creating true ownership of change was noted to be particularly powerful in ensuring consistent application of the change.

Informing large and complex PICU teams (iPARIHS *context*) of a change was identified as a challenge but was also noted to be vital to elevate awareness and ensure that teams worked in concert to use new processes. Multiple modalities of communication (both written and verbal) were needed within multiple settings (meetings, huddles, newsletters, email announcements, computer-based learning modules, etc.) to ensure reach to all providers. Finally, nurses, in particular, preferred advanced notice about upcoming change to allow them to prepare to integrate changes into workflow; they appreciated opportunities to practice using new skills or techniques in a simulated setting prior to roll-out.

Providers emphasized the need to have easy access to information and tools required for change at all times (iPARIHS *innovation*, *context*). Different units had various established options (electronic, hard copy, signage), but providers differed in their opinions about which of these worked best. Because multi-professional education about change was often completed at a time remote from actual implementation, additional “just-in-time” training at the time of change “roll-out” was viewed as very helpful, particularly by nurses.

Following initial implementation efforts, providers needed consistent and frequent exposure to a new intervention to ensure that it became part of their workflow (iPARIHS *innovation*). Infrequent opportunities to use a change (i.e., for a rare condition) made it difficult to integrate into practice, as one provider noted: “Sometimes everybody agrees that there’s going to be a policy change but it may be six months before you encounter a situation where that change would be implemented and then nobody remembers.” (Site 2 PICU, medical sub-specialist 18). Any effort to make change automatic or efforts to allow providers to practice using a change to create “muscle memory” around the change were also valuable.

### Sustainability of change in the PICU

Following initial implementation, sustainability of change in the PICU was a formidable challenge, but of significant interest, as maintaining the benefits of successful practice modifications over time is important. While iPARIHS does not address sustainability directly, we inferred identified strategies for sustainability through discussions of the iPARIHS *context* subconstruct “mechanisms for embedding change.” Aspects of planning, including ensuring buy-in from all professional groups, and promoting aspects of changes that have direct value for providers, were identified as being important for ultimate sustainability. While all provider roles saw great value in tracking and providing data around the impact of change, physicians in particular saw great benefit in this method to sustain buy-in and increase compliance. Importantly, many providers noted that their unit lacked resources to collect and report data back to unit staff, representing a major limitation to sustainability, and an opportunity for improvement. One attending physician involved in quality improvement commented: “There’s certainly great efforts to make more of our interventions data driven, but that hasn’t been emphasized as much…So creating that whole infrastructure – it’s a big task” (Site 1 CVICU, Attending 1). Providers also felt that frequent reminders about the rationale for change and about the change itself promoted sustainability.

### A model for implementing change in the PICU

**Fig. 1:**
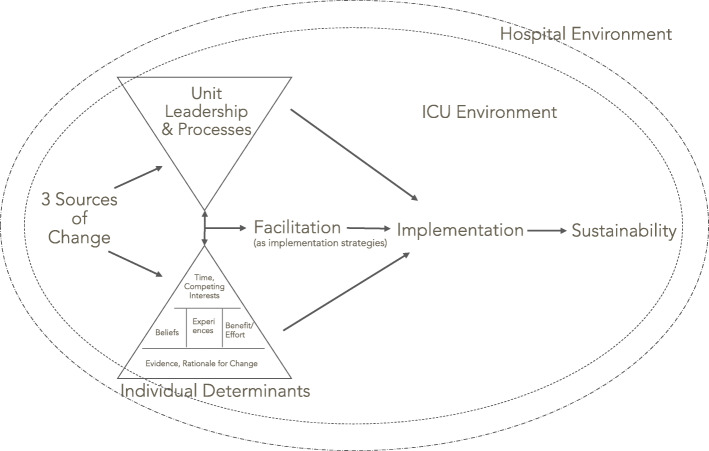
Model for implementing change in the PICU

Through exploration of the iPARIHS framework’s constructs and identification of the themes described above, we developed a model for change implementation in the PICU (Fig. 1). This model reflects the iPARIHS constructs and subconstructs and extends beyond iPARIHS to illustrate the relationships between PICU-specific factors that influence change, including the hospital and PICU environment that influence change appreciably. These relationships are not reflected in iPARIHS, but likely crucial to understand when planning implementation in the PICU. As a process model [31], our model describes the manner in which research is translated into practice within the PICU; this process includes multiple phases.

Ideas are initially proposed by individuals or groups (three sources of change, as described in Section 3.2). The decision to pursue change in the unit is made by unit leadership (unit leadership: Section 3.3). Our interviews did not explore the factors that influence how leadership selects changes to pursue; however, available unit resources, unit needs and priorities, and mandates at the hospital level likely contribute to these decisions. Individual providers also choose to support or resist a change based on multiple factors (individual determinants, Section 3.4), including the evidence and rationale for the change, their own beliefs and experiences, the potential benefit versus the effort required to change, and time required in light of other competing interests. While initial subthemes such as evidence and rationale for change are necessary, they are superseded by subsequent subthemes. As such, regardless of the evidence or rationale for a change, implementation will not occur unless providers perceive that the benefits of a change exceed the effort required and they have adequate time to operationalize the change. Change subsequently requires facilitation (Section 3.5), implementation, and later, efforts to ensure sustainability (Section 3.6).

Individual determinants and unit/team processes interact with one another and influence the selection of facilitation activities which are operationalized primarily as implementation strategies; likewise, facilitation activities impact these factors and are linked in a bidirectional fashion. Facilitation efforts enable implementation; whether or not a change is implemented is dependent on individual determinants and unit leadership and processes. This relationship is unidirectional. Finally, after initial implementation, if a change has been integrated into workflow, it may be sustained. The PICU environment exerts an influence on this entire process of change, impacting how individuals perceive proposals to change and their ability to integrate changes into standard work. To a lesser degree, the hospital may also influence change, often when hospital-wide modifications to practices are also mandated in the PICU. We acknowledge that this model is idealized in the linear arrangement; in actual practice, implementation steps are unlikely to be sequential, and implementation efforts may stall and require teams to go back to a previous step before a change is integrated into workflow. This model emphasizes the factors and their relationships that impact implementation and may be used for implementation planning in this setting.

## Discussion

From this qualitative study using the iPARIHS framework, we identified interactions between constructs and shaped those into a model for change in the PICU, which, to our knowledge, has not been previously described. Elements missing from the iPARIHS framework, but relevant in other implementation models [32, 33] have been added. Implications for planning implementation in the PICU exist within each model element and phase. The phases of change are shaped by the PICU environment, with an essential need to involve members of large, complex multi-professional teams, and acknowledge the high-risk, high-stakes nature of the work at near capacity, and apprehension about potentially worsening outcomes that often makes providers hesitant to improve care that is adequate, but could still be improved significantly. Decisions to change were made both by unit leadership who supported change at the unit level and individual providers who made an independent decision to support or resist changes. Some providers readily embraced change, while others sought out stability in this unpredictable environment. Notably, providers who were sometimes identified as the least likely to embrace change were also most influential in supporting initiatives and critical for ensuring buy-in and success; specifically involving these providers in change and ensuring their participation may be useful for implementation among other staff looking to these individuals for guidance around use of new changes. Sinuff et al. reported barriers and facilitators in relation to guideline adherence in adult ICUs and identified several findings similar to those in the PICU, notably the need for effective leaders, tailored education based on learning preferences, reminders, and data-based evaluation [16]. While Sinuff et al. stress the importance of creating a culture that supports guideline use [16], this study highlights some of the barriers in the PICU environment and culture that can hinder implementation and ultimately are important to address when creating an implementation plan.

### Using iPARIHS to understand PICU implementation efforts

The iPARIHS framework was generally helpful as a determinant framework in exploring implementation; we found the *innovation*, *recipients*, and *context* constructs overall were a useful scaffold to structure thinking about implementation in the PICU. Harvey and Kitson place *facilitation* central to the process of implementation in iPARIHS, as it “activates implementation through assessing and responding to characteristics of the innovation and recipients within their contextual setting” [17]. While the other main iPARIHS constructs are expanded for users with detailed subconstructs, facilitation is somewhat under-defined. The characteristics of a facilitator are described; however, the actions that constitute facilitation are not detailed, and there is no sub-classification of the types of activities or strategies that facilitators might utilize to enable and support change. Lack of clarity around the action of facilitation constituted somewhat of a “black box” in the framework, despite the fact that it remains crucial for implementation. We aimed to further describe facilitation by identifying activities that “activated implementation” through our interviews and subsequent qualitative analysis. While this work does not go as far as to propose a comprehensive set of facilitation subconstructs, we provide insight into facilitation activities that may be useful in the PICU, which are presented in Table 2. A strength of our work is the delineation of the activities important for facilitation, which in this PICU environment consisted primarily of activities that could be linked with known implementation strategies. By developing a more comprehensive and clear understanding of the facilitation construct, we aim to advance the framework and potentially inform future identification of important facilitation subconstructs within iPARIHS. We offer this critique based on our qualitative data and suggest that to further improve the framework, a more detailed description of components or features of facilitation would be useful for framework operationalization and in determining where facilitation barriers exist. Moreover, identifying these activities may be key to informing the selection of implementation strategies, which continues to be an active area of inquiry within implementation science [34] and holds practical value to those translating evidence into practice.

Implementation strategies identified through assessment of facilitation in our work in the PICU have been described by others in similar ICU environments. de Vos et al. and Stevens et al. noted the importance of engaged staff and adequate resources to facilitate change [5, 15]; in our study, facilitation of change was impacted by variable provider engagement and the time and personnel allocated to work on change. Other authors have reported the utility of some of the same facilitators in the ICU environment including the need for multiple educational modalities and a team of motivated champions [7, 8].

The process model that we have put forth organizes the iPARIHS constructs to reflect how change occurs in the PICU setting and potentially informs how groups might identify barriers and facilitators and plan implementation strategies. We did, however, find there were iPARIHS subconstructs that were infrequently discussed by providers (i.e., *goals*, *organizational absorptive capacity*) and may not be as important in this setting. We also identified novel concepts not included in the framework and interplay between constructs that had important implications for implementation.

Some of the iPARIHS subconstructs were difficult to separate from one another in the PICU setting. Within the *recipients* construct, *motivation* and *values and beliefs* were often inter-related. Within *innovation*, *usability* and *clarity* were related, and within *context*, we found that discussions of *past experiences with innovation and change* were linked closely to *mechanisms for embedding change*, as many efforts attempted to integrate changes into PICU workflow. While this did not complicate our analysis, the fact that these and other sub-constructs often “traveled together” or were interlinked should be noted.

There were a few frequently discussed concepts that did not easily fit within any of the iPARIHS subconstructs. These findings may reflect the fact that we created a process model from the determinant iPARIHS framework, but could potentially be considered useful additions to the framework. Providers’ *emotions* were conceptually distinct from *motivation* or *values and beliefs* and had an important influence on the work environment and the stressors that might prevent acceptance of change. Another example was the importance of having regular opportunities to use a change in actual or simulated practice for nurses; however, there was no subconstruct within *innovation* that captured this concept. We observed a few instances where factors within one construct significantly influenced another. For example, the concept of loss of autonomy with standardization of care fit within the construct of the *innovation*; however, *recipients* were also impacted. In examples like these, factors influencing implementation in the PICU did not fit neatly within single iPARIHS constructs; understanding the links between constructs has implications for implementation planning.

The iPARIHS framework lacks formal subconstruct definitions. Guided by other existing frameworks, we developed more explicit subconstruct definitions that may be used in future studies to guide a standard approach to qualitative analysis using this relatively new framework (Additional file 3). To further build on iPARIHS, we posit that standard definitions for subconstructs be developed to ensure consistent use of terms. This is particularly important for use of the framework within the research setting, as the need for uniform definitions has been noted frequently in implementation science [35]. We also suggest that further exploration of the concept of facilitation, with potential for development of subconstructs to further define components or actions. This will provide users with context to evaluate and operationalize plans around this critical element of the framework.

### Limitations

An aim of the larger study was to investigate implementation of blood transfusion recommendations, as such, we enrolled participants who ordered or administered transfusions but did not include other providers who may play a role in implementation of other PICU practice changes (respiratory therapists, pharmacists, patients, etc.). While it was not within the scope of this project to include the perspectives of all allied health professionals who work in the PICU, we perceive that our model of change, which is based on a number of different pediatric ICU models, is likely fairly comprehensive, but which may benefit from additional nuance that the inclusion of these groups might offer in future studies. While we aimed to ensure that PICUs were representative of the scope of US practice, it is possible that not all variations in PICU culture are represented and that the data presented is not fully generalizable to other PICUs; however, using the iPARIHS framework helped to ensure that we captured characteristics of the change process that are broadly transferable across settings. Barriers, facilitators, and implementation methods noted by interviewees reflect their opinions; in reality, different elements of the PICU setting may be more important to optimize implementation; however, this analysis serves as a starting point for guiding and testing implementation in this setting.

## Conclusions

The PICU environment and providers significantly shape the nature and process of implementing clinical practice changes, with need for accommodation of large, complex teams, high stakes work, and individual providers who approach change in light of their own experiences, beliefs, and capacity to integrate changes into an already demanding workflow. Unit or hospital-based systems may not provide resources to optimize facilitation and participation in change and often do not have established methods to provide data on the impact of changes. The iPARIHS framework was adequate to identify barriers and facilitators of change in this environment; however, further elaboration of subconstructs of facilitation was necessary to operationalize the framework. Our process model for implementation in the PICU may guide selection of implementation strategies in similar environments and augment efforts to integrate change into workflow.

## Supplementary Information

**Additional file 1.**

**Additional file 2.**

**Additional file 3.**

## Data Availability

The datasets used and/or analyzed during the current study are available from the corresponding author on reasonable request.

## Abbreviations

CVICU: Cardiovascular intensive care unit
PICU: Pediatric intensive care unit
ICU: Intensive care unit
iPARIHS: Integrated Promoting Action on Research Implementation in Health Services
NP: Nurse practitioner
PARIHS: Promoting Action on Research Implementation in Health Services
