# Supplementary material for: Implementation of clinical practice changes in the PICU: a qualitative study using and refining the iPARIHS framework
Source: Implement Sci. 2021 Jan 28;16:15. doi: 10.1186/s13012-021-01080-9 (PMC7841901; doi:10.1186/s13012-021-01080-9)
Supplement: Supplementary file 1 — Additional file 1. [file 13012_2021_1080_MOESM1_ESM.docx]

Standards for Reporting Qualitative Research (SRQR)

<https://www.equator-network.org/reporting-guidelines/srqr/>

| Number | Topic | Item | Description |
| --- | --- | --- | --- |
| Title and Abstract | |  |  |
| S1 | Title | Concise description of the nature and topic of the study identify the study as qualitative or indicating the approach or data collection methods. | The qualitative nature of the study as well as topic and intention of the study are included in the title. |
|  | Abstract | Summary of key elements of the study using the abstract format of the intended publication. | The study and key findings are summarized using the abstract format appropriate for Implementation Science. |
| Introduction |  |  |  |
| S3 | Problem Formulation | Description and significance of the problem/phenomenon studied; review of relevant theory and empirical work; problem statement | This is detailed in the Background section; relevant studies detailing implementation efforts in the ICU are reviewed along with rationale behind selection of the implementation framework used for this study. The problem statement “no study has attempted to characterize unique PICU barriers and facilitators” is noted in the third paragraph of this section. |
| S4 | Purpose or research question | Purpose of the study and specific objectives or questions. | The purpose of the study is to “address a gap in the literature around PICU-specific implementation barriers and facilitators using the i-PARIHS framework and puts forth a comprehensive process model to understand change in the PICU” |
| Methods |  |  |  |
| S5 | Qualitative approach and research paradigm | Qualitative approach and guiding theory if appropriate; identifying the research paradigm is also recommended; rationale | The framework approach to qualitative analysis as well as a brief description of this approach are included in the Methods section. The rationale behind the selection of iPARIHS framework as a basis for the qualitative analysis is stated. |
| S6 | Researcher characteristics and reflexivity | Researchers’ characteristics that may influence the research, including personal attributes, qualifications/experience, relationship with participants, assumptions, and/or presuppositions; potential or actual interaction between researchers’ characteristics and the research questions, approach, methods, results, and/or transferability | All researchers have been included as authors on this manuscript, and as such, their affiliations are listed. The authors who completed the interviews were both familiar with care in the PICU which was a benefit in establishing trust and familiarity with topics discussed by interviewees. The qualitative analysis team was composed of one research familiar with the ICU, one other medical sub-specialist and one qualitative researcher familiar with the medical system through prior research but without formal medical training. These three individuals were felt to be necessary to review, compare, contrast, and summarize data given their different perspectives. |
| S7 | Context | Setting/site and salient contextual factors; rationale | “We conducted interviews with health care providers working in various roles from eight PICUs across the US. We selected units to represent variation in PICU types (pediatric ICU (excluding cardiac patients, 4 units), pediatric cardiovascular ICU (CVICU) (2 units), and combined pediatric/cardiovascular PICU/CVICU (2 units)) and overall PICU size (11-32 beds) in the US.” |
| S8 | Sampling strategy | How and why research participants, documents, or events were selected; criteria for deciding when no further sampling was necessary; rationale | Participants functioned in various roles within the PICU: “PICU attendings (n=15), fellow trainees (6), resident trainees (4), nurse practitioners (NPs) or physician assistants (9), nurses (10), and subspecialty physicians/surgeons whose patients were cared for regularly in a PICU (6), including hematologists/oncologists, cardiologists and general and cardiothoracic surgeons. Providers in each role were interviewed at each site, with the exception of fellow or resident trainees, as they were not present in some units”. To determine when no further sampling was necessary: “We continued interviewing participants in each role until no new information was forthcoming from new participants. The qualitative team members (KS, LH, and GL) held meetings to review data and confirm when variation in responses was no longer noted.” |
| S9 | Ethical issues pertaining to human subjects | Documentation of approval by appropriate ethics review board and participant consent, or explanation for lack thereof; other confidentiality and data security issues | “Ethics approval for the study was obtained from the Stanford University Institutional Review Board (IRB-47140).” And “Participant’s verbal consent was obtained prior to each interview...” |
| S10 | Data collection methods | Types of data collected; details of data collection procedures including start and stop dates of data collection and analysis, iterative process, triangulation of sources/methods, and modification of procedures in response to evolving study findings; rationale | “Semi-structured qualitative interviews were carried out as part of a larger study to assess for prospective implementation of new blood transfusion recommendations for critically ill children. The aim of the interviews was to better understand implementation of clinical practice changes within the PICU as well as provider views on blood transfusion to later inform implementation of a set of new transfusion recommendations. This paper focuses on experiences with implementing general clinical practice changes in the PICU, and is not specific to blood transfusion.” “One of two authors (KS and MF) conducted interviews either in person or via telephone…Interviews were audio recorded and transcribed verbatim.” “[Interviews] were carried out between December, 2018 and June, 2019.” There were no modifications to the data collection procedures during the study. |
| S11 | Data collection instruments and technologies | Description of instruments and devices used for data collection; if/how the instruments change over the course of the study | The same interview topic guide was used for all subject interviews. “The interview topic guide was developed from the iPARIHS framework [15](Harvey, 2016) with additional topics that focused on barriers and facilitators of clinical practice changes and blood transfusion in the intensive care unit (Supplemental file 1). Participants were asked first to describe a clinical practice change that had been implemented in their ICU as a starting point to discuss the process and impact of these initiatives. Additional questioning focused on exploring PICU culture, receptivity to clinical practice change, initiating and sustaining practice changes, and experiences and perspectives on blood transfusion in the PICU. Qualitative data related to blood transfusion is not reported in this manuscript. The topic guide is included as Supplemental file 1. |
| S12 | Units of study | Number and relevant characteristics of participants, documents, or events included in the study; level of participation | “50 health care providers were interviewed: PICU attendings (n=15), fellow trainees (6), resident trainees (4), nurse practitioners (NPs) or physician assistants (9), nurses (10), and subspecialty physicians/surgeons whose patients were cared for regularly in a PICU (6), including hematologists/oncologists, cardiologists and general and cardiothoracic surgeons. Providers in each role were interviewed at each site, with the exception of fellow or resident trainees, as they were not present in some units”. |
| S13 | Data processing | Methods for processing data prior to and during analysis, including transcription, data entry, data management and security, verification of data integrity, data coding, and anonymization/deidentification of excerpts | “Interviews were…transcribed verbatim”. Transcribed interviews were anonymized and then coded using NVivo (Version 12) and a framework approach. Coded data was summarized along thematic categories structured around the iPARIHS constructs and subconstructs.” All quotes are reported in a manner that identifies the ICU type and role, however subjects are not identified. |
| S14 | Data analysis | Process by which inferences, themes, etc. were identified and developed, including the researchers involved in data analysis; usually references a specific paradigm approach; rationale | “We used a Framework Approach [20](Ritchie, 2003) for qualitative analysis with the NVivo (Version 12) software package. Framework is an approach to analysis in which case-level data (rows) are summarized along thematic categories (columns) in a matrix and involves five steps of familiarization: identifying a thematic framework, indexing, charting, and mapping and interpretation [21](Ritchie and Spencer, 1994). The coding framework was structured around the iPARIHS constructs (innovation (the evidence-based intervention), recipients, context, and facilitation) with associated subconstructs [15](Harvey, 2016). Qualitative analysis team members included KS, LH and GL (all authors). |
| S15 | Techniques to enhance trustworthiness | Techniques to enhance trustworthiness and credibility of data analysis; rationale | “At the outset of the analysis, two researchers (KS, LH) selected and coded fived interviews independently using the a priori codes from iPARIHS, while creating new codes for emergent themes. The researchers met to agree on definitions and interpretations of existing codes, compare coding, and discuss emerging themes and integrate into the coding framework. One researcher (KS) coded the remainder of the interviews using the established coding strategy.” And “Strategies to ensure credibility (internal validity) of findings followed guidance by Miles et al (2019) and included linking data to categories in iPARIHS, checking for negative evidence, and checking that findings are replicable across the dataset (i.e., across more than one PICU).” |
| Results/findings |  |  |  |
| S16 | Synthesis and interpretation | Main findings; might include development of a theory or model, or integration with prior research or theory | The manuscript puts forth a model for implementing change in the PICU (section 3.7) that is based on exploration of the iPARIHS constructs. |
| S17 | Links to empirical data | Evidence to substantiate analytic findings | Quotes are provided throughout the manuscript as well as in both tables. Additionally, the datasets used are readily available from the corresponding author upon request. |
| Discussion |  |  |  |
| S18 | Integration with prior work, implications, transferability, and contributions to the field | Short summary of main findings; explanation of how findings and conclusions connect to, support, elaborate on, or challenge conclusions of earlier scholarship; discussion of scope of application/generalizability; identification of unique contributions to scholarship in a discipline or field | The Discussion section summarizes main findings and links these findings to existing literature. Additionally, we review aspects of the iPARIHS framework that were useful and indicate potential areas in which it may be built upon to enhance utility in clinical settings such as the PICU. Unique contributions are also highlighted in the “Contributions to the Literature” section and the beginning of the manuscript. |
| S19 | Limitations | Trustworthiness and limitations of findings | Limitations are detailed in section 4.2 and relate to representativeness of providers sampled in the PICU as a whole as well as the representativeness of the units nationally. Additionally, we point out that interviews reflect individuals’ opinions and that other barriers and facilitators not identified may impact implementation. |
| Other |  |  |  |
| S20 | Conflicts of interest | Potential sources of influence or perceived influence on study conduct and conclusions; how these were managed | “The authors declare that they have no competing interests” |
|  | Funding | Sources of funding and other support; role of funders in data collection, interpretation, and reporting | “Research reported in this publication was supported by the National Heart, Lung, and Blood Institute of the National Institutes of Health under Award Number K12HL137942.” The funders had no direct role in data collection, interpretation or reporting. |

|  | Reference:  O'Brien BC, Harris IB, Beckman TJ, Reed DA, Cook DA. **Standards for reporting qualitative research: a synthesis of recommendations.** *Academic Medicine*, Vol. 89, No. 9 / Sept 2014 |
| --- | --- |
