# Supplementary material for: Implementation of clinical practice changes in the PICU: a qualitative study using and refining the iPARIHS framework
Source: Implement Sci. 2021 Jan 28;16:15. doi: 10.1186/s13012-021-01080-9 (PMC7841901; doi:10.1186/s13012-021-01080-9)
Supplement: Supplementary file 2 — Additional file 2. [file 13012_2021_1080_MOESM2_ESM.docx]

**Provider interview guide**

| CHANGE AND IMPACT OF CHANGE IN THE ICU  Before we ask questions about blood transfusion, I want to ask you some general questions about how change occurs in your ICU. Are you ready to get started? | |
| --- | --- |
| Core Question | Supplemental Questions |
| 1. Tell me about a [clinical] change in practice that has recently occurred where you work. | - Who proposed the change?  - How was this proposal made?  - Who were the thought leaders who drove the change?  - Who usually has a “say” in terms of change?  - How did you find out about the change? What are the most effective ways people find out about change in your unit?  - How did this change occur in your unit?  - Who enacted the change?  - What methods were used to facilitate change?  - What are the usual barriers to change?  - What types of things help change occur?  - What was your role in the change?  - Do you feel like you have a role/ownership in any changes that occur?  - What went well with this change?  - How could it have been improved?  - What makes change successful or unsuccessful?  - What makes change “stick” in your unit?  - Does the hospital facilitate change/innovation? How? |
| 1. How do you typically respond to change? | - What makes change easier?  - What makes change more difficult?  - Accountability for change (self/peers/supervisor/institution)  - How do you see others respond to change? Receptive to changing practice? |
| 1. If you had a concern or problem related to your work in the unit, who would you discuss it with? | - peers, leadership, administration?  - To what extent do you feel supported?  - Who can you depend on for support?  - Who do others (outside role/group) depend on? |
| 1. How do you know you’ve done a good job at work? | - How do you find out if there are areas where you need to improve?  - What is this experience like?  - How do others get feedback about their work? |
| Thank you very much for that information. Is there anything I haven’t asked about that would be important for me to understand about the working environment in the ICU? | |
| *Note: Questions about blood transfusion decision making and attitudes around restrictive transfusion, contextual factors around implementation of a set of blood transfusion guidelines, and provider opinions about computerized decision support tools followed these questions, but are not reported in this manuscript.* | |
