# Supplementary material for: Implementation of clinical practice changes in the PICU: a qualitative study using and refining the iPARIHS framework
Source: Implement Sci. 2021 Jan 28;16:15. doi: 10.1186/s13012-021-01080-9 (PMC7841901; doi:10.1186/s13012-021-01080-9)
Supplement: Supplementary file 3 — Additional file 3. [file 13012_2021_1080_MOESM3_ESM.docx]

**Codebook: iPARIHS Subconstruct Definitions**

| Major Construct | Sub-Construct | Definition |
| --- | --- | --- |
| Innovation | | |
|  | Underlying knowledge sources | Specified sources of information relevant to the specific innovation, including research/published guidelines, clinical/patient experience, and/or local practice information (1) |
|  | Clarity | How well the innovation is understood by the recipients |
|  | Degree of fit with existing practice and values | How an EBP aligns with individuals’ or group’s own norms, values, and perceived risks and needs; and how an EBP fits with existing workflows and systems (1, 2) |
|  | Usability | The extent to which an innovation is able to be used in the work system. |
|  | Relative advantage | Stakeholders’ perception of the advantage [or disadvantage] of implementing the EBP versus an alternative solution; or stakeholders’ cost-benefit perceptions compared to other alternatives. (1, 2) |
|  | Trialability | The ability to test the EBP on a small scale in the organization, and to be able to reverse course (undo implementation) if warranted. (1, 2) |
|  | Observable Results | The degree to which the results of an innovation are visible or able to be seen being used in practice by adopters. (1, 2, 3) |
| Recipients | | |
|  | Motivation | The reason(s) one has for acting or behaving in a particular way OR the general desire or willingness of someone to do something. |
|  | Values and beliefs | Individual or group principles or standards of behavior, ideas determine Rogers Theory of Diffusion Innovation, d to be “true”, or what is determined to be important. (4) |
|  | Goals | The end state toward which an individual or group is striving. (4) |
|  | Skills and knowledge | Abilities or proficiencies that are obtained through training/practice and the awareness of specific facts or concepts. (4) |
|  | Time, resources, support | Things or human resources that are used to achieve a goal, and anything that aids or assists in achieving a goal (4) |
|  | Local opinion leaders | Individuals who are seen as likable, trustworthy, and influential. These individuals may be able to persuade others to implement a new innovation through their influence. (5) |
|  | Collaboration and teamwork | Cooperative effort toward a common goal or on a common project. (4) |
|  | Existing networks | The nature and quality of webs of social networks within an organization. (2) |
|  | Power and authority | The capacity or power to influence events, behaviors, situations or people, even when others try to resist this influence. (4) |
|  | Presence of boundaries | The presence of barriers to sharing knowledge and spreading work practices. (6) |
|  | Expectations or Requirements: ADDED to iPARIHS by authors | Tasks, engagement/participation in efforts that people are expected or required to complete for their job. Use: when there is a disconnect between individuals or groups that vary in their ideas of what is expected or required. |
|  | Feelings or Emotions: ADDED to iPARIHS by authors | Any feeling or emotion a recipient might have about something related to work, implementation efforts, or the innovation itself |
| Context |  | The physical environment/setting, as well as the boundaries or structures that shape the setting. (1) |
| Local Level | | |
|  | Formal and informal leadership support | The behaviors, attitudes and actions of leaders that reflect readiness or receptivity to a change. (1) |
|  | Culture | Prevailing norms, values, and basic assumptions of a given organization, or “the way things are done around here”. (1, 2) |
|  | Past experience of innovation and change | Prior experience with the introduction of something new. |
|  | Mechanisms for embedding change | The process of incorporating a new thing or process such that it is fully implemented and consistently used in day-to-day work. |
|  | Evaluation and feedback processes | The systems/processes for collecting, displaying and using EBP–related data to stakeholders in meaningful forms to measure practice or change. (1) |
|  | Learning environment | A climate in which: a) leaders express their own fallibility and need for team members’ assistance and input; b) team members feel that they are essential, valued, and knowledgeable partners in the change process; c) individuals feel psychologically safe to try new methods; and d) there is sufficient time and space for reflective thinking and evaluation. (2) |
| Organizational Level | | |
|  | Organizational priorities | Values and goals that guide the actions of an organization |
|  | Senior leadership and management support | The behaviors, attitudes and actions of hospital leaders outside and above the ICU that reflect readiness or receptivity to a change. |
|  | Culture | Prevailing norms, values, and basic assumptions of a given organization, or “the way things are done around here”. (1, 2) |
|  | Structure and systems | The social architecture, age, maturity, and size of an organization. (2) |
|  | History of innovation and change | Prior experience with the introduction of something new. |
|  | Absorptive capacity | The capacity for change, preparedness, and receptivity of involved individuals to an innovation, and the extent to which use of that innovation will be rewarded, supported, and expected within their organization. (1, 2) |
|  | Learning networks | Continuing education, collaboratives, or other organized efforts to enhance an individual or group’s knowledge. |
| Facilitation |  | A deliberate process of interactive problem solving and support that occurs in the context of a recognized need for improvement and a supportive interpersonal relationship (1) |

New Nodes

| Consistent Exposure to Intervention | The idea that implementation is easier or more sustainable when individuals have the need/opportunity use the intervention frequently. |
| --- | --- |
| Provider Autonomy | Self-directed and independent activity by a care provider (in decision making, etc.). Usually in the context of a provider discussing the value of autonomy, fear of losing autonomy. |
| Response to bad outcome | Quality or improvement efforts that occur in response to events where a negative outcome occurred. |
| Standardization of Practice | Ideas or opinions (good or bad) relating to consistency of medical practice between patients or over time |

References:

1. Stetler CB, Damschroder LJ, Helfrich CD, Hagedorn HJ. A guide for applying a revised version of the PARIHS framework for implementation. Implement Sci. 2011;30:6.
2. Damschroder LJ, Aron DC, Keith RE, Kirsh SR, Alexander JA, Lowery JC. Fostering implementation of health services research findings into practice: a consolidated framework for advancing implementation science. Implement Sci. 2009;4:50.
3. Kislov R, Walshe K, Harvey G. Managing boundaries in primary care service improvement: A developmental approach to communities of practice. Implement Sci. 2012;7:97.
4. Rogers EM. Diffusion of Innovations. 3^rd^ ed. New York: The Free Press; 2003.
5. Flodgren G, Hall AM, Goulding L, Eccles MP, Grimshaw JM, Leng GC, et al. Tools developed and disseminated by guideline producers to promote the uptake of their guidelines. Cochrane Database Syst Rev. 2016;(8): CD010669. doi: 10.1002/14651858.CD010669.pub2.
6. Cane J, O’Connor D, Michie S. Validation of the theoretical domains framework for use in behaviour change and implementation research. Implement Sci. 2012;7:37.
